# THE RISK OF DEMENTIA AMONG INDIVIDUALS WITH DEPRESSION AFTER A TRAUMATIC BRAIN INJURY

**DOI:** 10.1093/geroni/igac059.1811

**Published:** 2022-12-20

**Authors:** Joan Ashby, Miriam Nuno, Beatrice Ugiliweneza, Maya Deshmukh, Maxwell Boakye

**Affiliations:** University of California, Davis/Davis, Davis, California, United States; University of California, Davis/Davis, Davis, California, United States; Univerisity of Louisville, Louisville, Kentucky, United States; University of California, Davis/Davis, Davis, California, United States; Univerisity of Louisville/Louisville, Louisville, Kentucky, United States

## Abstract

Although traumatic brain injury (TBI) has been found to be associated with dementia and depression, large‐scale studies of US non‐Veteran populations are limited. This study assesses the role of depression on dementia risk after TBI. Data was analyzed from 80,423 individuals with TBI age 55+ in the IBM MarketScan Commercial Claims and Encounter Database and the Medicare Supplemental Database between January 1, 2000 and December 31, 2019. Dementia diagnosis was captured from inpatient or outpatient visits with a “wash out” period of one year. Depression was defined based on documented diagnosis and/or the prescription of antidepressants. The median age was 69 years, 51% female, with a majority covered by Medicaid (51%). 62% of TBIs were moderate/severe and 16% mild. 44,234 (55.1%) individuals were diagnosed with depression with a median of 2.1 months after TBI and 24.4 months prior to dementia. Depression rates differed by sex (female: 54%, male: 44%, p< 0.0001) and insurance (Commercial: 36%, Medicaid: 49%, p< 0.0001). The median time to dementia was 158 months. The median time to dementia was 128 months among those with depression, while patients without depression did not reach this estimate within 250 months of follow-up (p< 0.0001). The risk of dementia increased significantly over time after depression among patients with TBI. This study provides evidence to support that depression after TBI represents a symptom of TBI rather than a prodrome of dementia. Future research will investigate the role of depression in the risk of dementia by race/ethnicity, insurance status, and TBI severity.

